# Identification of small molecules uncoupling the Notch::Jagged interaction through an integrated high-throughput screening

**DOI:** 10.1371/journal.pone.0182640

**Published:** 2017-11-03

**Authors:** Natalia Platonova, Chiara Parravicini, Cristina Sensi, Alessandro Paoli, Michela Colombo, Antonino Neri, Ivano Eberini, Raffaella Chiaramonte

**Affiliations:** 1 Department of Health Sciences, Università degli Studi di Milano, Milano, Italy; 2 Department of Pharmacy, Università degli Studi di Pisa, Pisa, Italy; 3 Department of Pharmacological and Biomolecular Sciences, Università degli Studi di Milano, Milano, Italy; 4 Department of Oncology and Hemato-oncology, Università degli Studi di Milano; Hematology, Fondazione Cà Granda IRCCS Policlinico, Milano, Italy; Danish Cancer Society Research Center, DENMARK

## Abstract

Notch signaling plays an important role in several cellular functions including growth, differentiation, cell fate determination and stemness. Increased Notch activity has been linked to several types of cancers. Activation of Notch signaling is triggered by the interaction of Notch receptors (Notch1-4) with 5 different ligands (Jagged1-2 and Dll1-3-4) expressed on the neighbouring cells. Currently, indirect approaches to inhibit Notch signalling are based on the inhibition of the key step of Notch activation catalyzed by the γ-Secretase and thereby affect several different γ-Secretase substrates; conversely direct strategies get advantage of antibody-based drugs. The evidence that Jagged-mediated Notch activation plays a key role in cancer cell biology and the interplay with the surrounding microenvironment prompted us to develop a strategy to directly inhibit Notch activation by uncoupling its interaction with the Jagged, using an unprecedented approach based on small molecules. We set-up a screening strategy based on: protein::protein docking of crystallographic structures of Notch1 with Jagged1; comparative modelling of the Notch2:Jagged2 complex, based on the Notch1::Jagged1 complex; *in silico* high-throughput screening directed to Notch2 interaction surface of a virtual chemical library containing a large variety of molecules commercially available. The predicted pharmacological activity of the selected compounds was validated i*n vitro* by a gene reporter and a viability assay. This approach led to the successful identification of two candidates with different anti-proliferative potency and efficacy. This represents the first step towards the rational identification of candidate molecules for the development of entirely novel drugs directed to inhibit Notch signaling in cancer.

## Introduction

Notch signaling is activated through cell-cell communication in several physiologic and pathologic processes as the result of the engagement of Notch receptors at cell surface by their ligands located on the neighboring cells. Mammals express four different receptor isoforms, named Notch1, 2, 3 and 4, and two families of ligands, Jagged1 and 2, and Delta-like (Dll) 1, 3 and 4. Upon ligand binding, two proteolytic cleavages release the intracellular Notch portion (ICN) which translocates to the nucleus and triggers the CSL (CBF1/Su(H)/Lag-1) transcription factor to transactivate the Notch target genes. Many of these genes are positive regulators of proliferation and survival and include c-Myc, Bcl2, HES1 [1], or are involved in the downstream regulation of anti-apoptotic/proliferative pathways such as AKT, NF-κB, Wnt and others [2]. Notch signaling has also been reported to play a crucial role in the regulation of stem cell and cancer stem cell renewal [3].

Alterations in Notch signaling cause wide modifications in cell biology and tissue development associated to cancer and genetic diseases. Hereditary diseases are determined by mutation in Notch-related genes resulting in reduced Notch signaling with consequent altered development such as Alagille syndrome, spondylocostal dysostosis [4–6] and cerebral autosomal dominant arteriopathy with subcortical infarcts and leukoencephalopathy (CADASIL) [7]. Oppositely, Notch-related cancers are more frequently associated to increased Notch activity, indicating an oncogenic role of Notch, although a tumor suppressor effect of Notch signaling has also been reported in a minor number of malignancies [2]. Notch signaling gain-of-function may be acquired by mutations in Notch receptors that, therefore, result constitutively activated independently from ligand engagement. This is the case of T-cell acute lymphoblastic leukemia (T-ALL) [8] and other tumors, including breast, ovarian, lung, prostate cancer [2]. On the other side, Notch signaling may be hyperactive although Notch activation still depends on ligand engagement. This occurs when Notch signaling is dysregulated due to i) increased copy number or hyperexpression of Notch receptors and ligands [9–12]; ii) altered expression of regulative elements, such as Skeletrophin that controls Jagged2 activity [13] or Fbw7 that negatively regulates the stability of ICN [14]; iii) constitutive activation of upstream pathways, i.e. NF-κB, AKT, Wnt, mTOR, or hypoxic and inflammatory tumor microenvironment, i.e. IL6, TNFα [15–17]. In all these cases, uncoupling the interaction between Notch and its ligands is a critical step to hamper Notch aberrant activation.

Current therapeutic approaches of Notch inhibition mainly rely on the use of γ-secretase inhibitors (GSIs) that indirectly block the activation of all four Notch receptors by inhibiting the enzymatic activity of the γ-secretase complex [11]. GSIs do not exclusively inhibit Notch activation, but affect more than 50 further γ-secretase substrates including amyloid precursor protein, E-cadherin, N-cadherin, LDL receptor-related protein, ErbB-4, etc [18]. Moreover, GSIs display important side effects on intestinal toxicity due to intestinal goblet cell metaplasia associated to pan-Notch I inhibition [19]. This prompted the development of novel selective and direct approaches to inhibit Notch activation. Up to now, the selectivity has been obtained developing antibody-based drugs directed against Notch receptors (i.e. Notch1, Notch2 and Notch3), and the Dll4 ligand [11].

This work aims at laying the basis of an unprecedented approach directed to uncouple the interaction of Notch receptors and ligands by using specific small molecules that offer the advantages of an oral administration route and no need of nosocomial admission with a consequent better patient’s quality of life and lower social costs. In particular, here we have directed our first effort to inhibit Notch2 activation trigged by the ligand Jagged2 on the basis of our interest in multiple myeloma, an hematological malignancy associated to Notch2/Jagged2 dysregulation. Multiple myeloma is a deadly disease still incurable in which the high levels of Notch signalling affects both tumor cell biology and the interactions with the surrounding microenvironment, inducing a pro-tumorigenic phenotype. We recently showed that Notch2 expression was significantly increased in myeloma patients carrying the high-risk translocations t(14;16)(q32;q23) and t(14;20)(q32;q11) resulting in Notch signalling activation [17]. This effect is attributable to Notch2 transactivation triggered by C-MAF and MAFB, the two transcription factors hyper-expressed by the two transclocations [20]. Notch2 activation may also be triggered by Dll1 on surrounding stromal cells resulting in the development of bortezomib resistance [21]. On the other side, Notch2 signalling triggered in osteoclast precursors by myeloma cell-derived Jagged ligands is key in activating tumor-associated osteoclast differentiation and bone destruction [22]. Concerning the Jagged2 ligand, the dysregulation is an early event occurring in the benign phase of monoclonal gammopathy of uncertain significance as a consequence of different genetic and epigenetic mechanisms [13,23] and play a key role in prompting bone marrow stromal cells to support myeloma cell growth by releasing key cytokines including IL-6, IGF-1, VEGF [17,24]. Finally, Jagged2 has been reported as critical for MM cell self-renewal *in vitro* and *ex vivo* [25].

In this study, based on an integrated high-throughput screening (HTS), we combine protein::protein molecular docking, homology modelling and a virtual screening of a large chemical library to select *in silico* a set of small molecules able to disrupt the Notch2::Jagged2 complex. The two top-scoring compounds were validated for their biological activity. This approach represents a successful strategy to identify small molecules which directly antagonize Notch signaling activation and display anti-proliferative effect.

## Materials and methods

### Notch2::Jagged2 molecular modelling

All the computational procedures were carried out using the suite Molecular Operating Environment (MOE), 2013.08 (Chemical Computing Group Inc., Montreal, QC, Canada).

In order to obtain a model of the complex between Notch1 and Jagged1, their crystallographic structures 2VJ3 and 2VJ2 (released on 2008-07-29, [26], respectively, were obtained from RCSB PDB (http://www.rcsb.org/pdb/home/home.do; 2014, January) and submitted to an automated procedure for correcting structures and preparing macromolecular data, through the MOE ‘Protein preparation’ program. The two proteins were docked, using the ZDOCK Sever version 3.0.2 (http://zdock.umassmed.edu/; 2014, January) [27] and driving the docking with some of the residues identified as relevant for the interaction between Notch and Jagged: i.e., Pro199, Arg201; Arg203; Asp205, Pro207 and Ile335 in Jagged with Val453 and Gly472 in Notch [26]. We analysed the 10 top-scoring complexes according to the ZDOCK score, and selected the ones recapitulating the experimental interactions. The Notch2::Jagged2 complex was built by comparative modelling on the Notch1-Jagged1 structures previously computed, through the MOE ‘Homology Model’ application, using default conditions. Briefly, as a first step, the primary structures of human Notch2 (Q04721) and Jagged2 (Q9Y219) were obtained from the UniProt database (http://www.uniprot.org/) and aligned against the sequences of Notch1 (P46531) and Jagged1 (P78504), respectively, with the MOE ‘Alignment tool’, in order to produce a reference alignment between the target sequences and the templates for the homology modelling procedure. Ten independent models of the main chain were built by the Boltzmann-weighted randomized modelling procedure, and one sidechain model was built for each one of these main chains using the unary quadratic optimization (UQO) procedure [28]. Each single intermediate model of the ensemble was submitted to a brief series of highly tethered energy minimizations (EMs), meant only to relieve steric strain, and scored according to the GB/IV scoring function [29]. On the top scoring model, the ionization states and proton placement were optimized with the Protonate 3D application, prior to the final refinement step through EM procedure to a RMS gradient value of 0.5 kcal/mol Å. In all the molecular mechanics procedures (MM), the Amber12:EHT forcefield was used.

### Notch2::Jagged2 molecular docking

An Asinex database of 438,407 commercial 2D compounds, providing diverse and cost effective coverage of drug-like chemical space, was selected (www.asinex.com). The majority of the included compounds have a high degree of drug-likeness, in accordance with Lipinski’s rule of 5. All the entries were *in silico* pre-processed with the MOE ‘Database Wash’ tool to correct systematic structure errors and badly-scaled bond lengths, add explicit hydrogen atoms, rebalance protonation states by deprotonating strong acids and/or protonating strong bases, remove explicit counter-ion and solvent structures, convert them in 3D and energy minimize their structures through the MOE ‘Database Minimize’ tool with the MMFF94x forcefield. All the rotatable bonds have been subsequently extensively explored through the MOE ‘Dock’ procedure as described below.

The dataset was tested *via* a well-established high throughput screening (HTS) protocol, performed only on the Notch2 surface, previously identified to interact with Jagged2. In order to speed-up the HTS procedure, we used a two-step docking protocol, in which each step was characterized by a different ability to prioritize compounds.

As a first step, the ‘Virtual Screening’ protocol, as implemented in the MOE ‘Dock’ application, was applied on the whole dataset of compounds, in which only the top-scoring solutions for each entry is submitted to the subsequent refinement steps. Briefly, all the rotatable bonds were explored for each compound, generating 20,000 conformers. The placement methodology was set to Triangle matcher, in which poses were generated by superposition of ligand atom triplets and triplets of receptor site points. The receptor site points were alpha sphere centers which represent locations of tight packing. At each iteration, a random conformation was selected. A random triplet of ligand atoms and a random triplet of alpha sphere centers were used to determine the pose. The maximum number of poses returned by each ligand placement was set to 1,000. These 1,000 poses were ranked according to the empirical scoring function London dG [30] and duplicated poses were removed.

In detail, according to the MOE ‘Docking’ documentation, the London dG scoring function estimates the free energy of binding of the ligand from a given pose. The functional form is a sum of terms:

$$\Delta G=c+E_{flex}+\sum_{h-bonds}c_{HB}f_{HB}+\sum_{m-lig}c_{M}f_{M}+\sum_{atoms\;i}\Delta D_{i}$$ (1)

where *c* represents the average gain/loss of rotational and translational entropy; *E*_*flex*_ is the energy due to the loss of flexibility of the ligand (calculated from ligand topology only); *f*_HB_ measures geometric imperfections of hydrogen bonds and takes a value in [0,1]; *c*_HB_ is the energy of an ideal hydrogen bond; *f*_M_ measures geometric imperfections of metal ligations and takes a value in [0,1]; *c*_M_ is the energy of an ideal metal ligation; and *D*_*i*_ is the desolvation energy of atom *i*. The difference in desolvation energies was calculated according to the formula

$$\Delta D_{i}=c_{i}R_{i}^{3}\{{∭}_{u\notin A⋃B}{\left|u\right|}^{-6}\;du-{∭}_{u\notin B}{\left|u\right|}^{-6}\;du\}$$ (2)

where *A* and *B* are the protein and/or ligand volumes with atom *i* belonging to volume *B*; *R*_*i*_ is the solvation radius of atom *i* (taken as the OPLS-AA van der Waals sigma parameter plus 0.5 Å); and *c*_*i*_ is the desolvation coefficient of atom *i*. The coefficients {c, cHB, ci} were fitted from approximately 400 X-ray crystal structures of protein-ligand complexes with available experimental pK_i_ data. Atoms were categorized into about a dozen atom types for the assignment of the *c*_*i*_ coefficients. The triple integrals were approximated using Generalized Born integral formulas.

The 25 top-scoring compounds, obtained from the initial ‘Virtual Screening’ protocol, were resubmitted to a second, more extensive docking procedure, consisting of two docking phases, placement and refinement. The first step was performed according to the previously described method, but, instead of keeping only one pose *per* compound, a higher number (30) of solutions were retained for each protein::ligand complex. Moreover, the 30 the top-scoring complex according to the London dG empirical scoring function were submitted to a further refinement step based on MM and the resulting complex was rescored according to the GBVI/WSA ΔG empirical scoring function, in order to obtain an approx. binding free energy value [30].

In detail, the refinement procedure was carried out considering the receptor as a rigid structure.

In order to speed up the calculation, residues over a 6 Å cutoff distance away from all the pre-refined poses were ignored, both during the refinement and in the final energy evaluation. However, if after the refinement any poses were moved to within 80% of the cutoff distance of any ignored atoms, the refinement stage was repeated with an increased cutoff distance.

According to the MOE ‘Docking’ documentation [31] the GBVI/WSA ΔG is a forcefield-based scoring function which estimates the free energy of binding of the ligand from a given pose. It was trained using the MMFF94x and AMBER99 forcefield on the 99 protein-ligand complexes of the SIE training set [30]. The functional form is a sum of terms:

$$\Delta G\approx c+\alpha \left[\frac{2}{3}({\Delta E}_{Coul}+{\Delta E}_{sol})+{\Delta E}_{vdW}+{\beta \Delta SA}_{weighted}\right]$$ (3)

where:

c represents the average gain/loss of rotational and translational entropy; α, β are constants which were determined during training (along with c and are forcefield-dependent; E_Coul_ is the coulombic electrostatic term which is calculated using currently loaded charges, using a constant dielectric of εi = 1; E_sol_ is the solvation electrostatic term which is calculated using the GB/VI solvation model. For more information on the GB/VI solvation model, please see Potential Energy Selection and Configuration; E_vdW_ is the van der Waals contribution to binding; SA_weighted_ is the surface area, weighted by exposure. This weighting scheme penalizes exposed surface area.

All the docking procedure were performed with the MOE ‘Dock’ module, and the Amber12:EHT forcefield.

For the two top-scoring compounds, the final ligand::protein complexes were optimized and their binding free energy values were computed through a collection of MOE procedures for interactive ligand modification, geometry optimization and MM-based energy minimization in the active site, namely LigX. In detail, during these calculations, the receptor atoms far from the ligand were held fixed, while receptor atoms in the active site were allowed to move but subjected to tether restraints to discourage gross movement. The ligand atoms were configured as free to move.

The binding site was automatically identified selecting the interacting residues on the Notch2::Jagged2 interface through the specific MOE ‘Protein contact’ tool. All the residues described by Cordle et al [26] and used for the guided Z-Dock procedure were included in this subset.

For summarizing the interactions between ligands and proteins, a fingerprint scheme was used, as implemented in the MOE software [32], in which interactions and surface contacts are classified according to the residue of origin, and built into a fingerprint scheme which is representative of the database of protein-ligand complexes.

A scheme summarizing the *in silico* HTS pipeline is reported in S1 Fig.

### Cell lines and reagents

The human MM cell lines, U266 (ATCC® TIB-196™) and HEK293 (ATCC® CRL-1573™) were purchased from the American Type Culture Collection, KMS12 were purchased from DSMZ collection of microorganisms and cell cultures. U266 and KMS12 were grown in RPMI1640 (Lonza, Italy) and HEK293T cell line was grown in DMEM (Lonza, Italy); both media were supplemented with 10% heat-inactivated FBS (Euroclone, Italy), 2 mM L-Glutamine (Euroclone, Italy), 100 U/ml Penicillin and 100 μg/ml Streptomycin (Sigma-Aldrich, Germany).

DAPT (GSI-IX, N-[N-(3,5-Difluorophenacetyl)-L-alanyl]-S-phenylglycine t-butyl ester) was purchased from Sigma-Aldrich, reconstituted in DMSO and administered to cells at a final concentration of 25 μM. Three compounds selected from the docking procedure, namely BAS 00693376, BAS 00327971 (named from here on as IGOR1 and IGOR2, respectively), and ASN13406980 (used as negative control), were purchased from Asinex (www.asinex.com) and reconstituted in DMSO at a concentration of 50 mM.

### Notch reporter assay

HEK293T cells were transiently transfected using Turbofect (Fermentas, Italy) with a firefly luciferase reporter plasmid carrying a multimerized 6xCSL (CBF1-RBP-J) Notch responsive element [33] and a plasmid carrying the Renilla luciferase under the control of thymidine kinase promoter (TK-pRL), which provides an internal control for transfection for normalization. Immediately after transfection, cells were treated with the selected compounds IGOR1 and IGOR2, DAPT or vehicle (DMSO) at the indicated concentrations. After 48 h of incubation, luciferase activity was measured in triplicate by Dual-Luciferase Reporter Assay System (Promega).

### Cell growth assay

MM cell lines were seeded in quadruplicate at 50,000 cells/ml in 96-well plate and treated with different concentrations of the compounds, DAPT or vehicle. DAPT treatment was repeated every 48 h. After 48 h and 96 h of incubation, cell growth was assessed by the volumetric count function for alive cells of the BD FACSVerse™ flow cytometer (BD Bioscience, Italy). Assays were performed in triplicate.

### Statistical analysis

Data are presented as mean values ± standard deviation (SD) of at least 3 independent experiments. Statistical analysis was performed by unpaired two-tailed Student’s t-test.

## Results and discussion

### *In silico* identification of small molecules disrupting Notch2::Jagged2 interactions

In order to identify novel small molecules able to inhibit the interaction between Notch2 and Jagged2, we implemented the following strategy: (i) protein::protein docking of crystallographic structures of Notch1 with Jagged1, guided through the residues known to be located at the Notch1::Jagged1 interface [26]; (ii) comparative modelling of the complex Notch2::Jagged2, built using the complex from (i); (iii) an *in silico* HTS directed to Notch2 interaction surface of a virtual chemical library containing a large variety of molecules, including very diverse chemical structures [34].

#### Molecular modelling of the Notch2::Jagged2 complex

We carried out the protein::protein docking procedure between Notch1 and Jagged1 by driving it through a set of experimentally identified and highly-conserved residues that have been demonstrated to be involved in the regulation of the disulphide-rich Delta/Serrate/LAG-2 (DSL) domain: Val453 and Gly472, for Notch1, and Phe199, Arg201, Arg203, Asp205, Phe207, for Jagged1 (details in Materials and Methods). The default ZDOCK set-up generates 2,000 solutions, and 1,868 out of these 2,000 were filtered out because of atom clashes.

The ten top-scoring solutions out of the 137 generated ones, according to the ZDOCK score [35] were carefully analyzed. Only two out of the ten docking solutions satisfied all the previous residue interactions and were characterized by a low root mean square deviation value (RMSD, 3.97 Å) after α-carbon superposition, and by the same potential energy value after minimization. The obtained supramolecular complexes allowed us to exactly identify the interaction surfaces relevant for the molecular recognition between Notch2 and Jagged2. Because of the degeneracy of the two selected complexes (potential energy: -3,384 versus -3,388 kcal/mol), just one of them was selected for further computational analyses.

**Fig 1:**
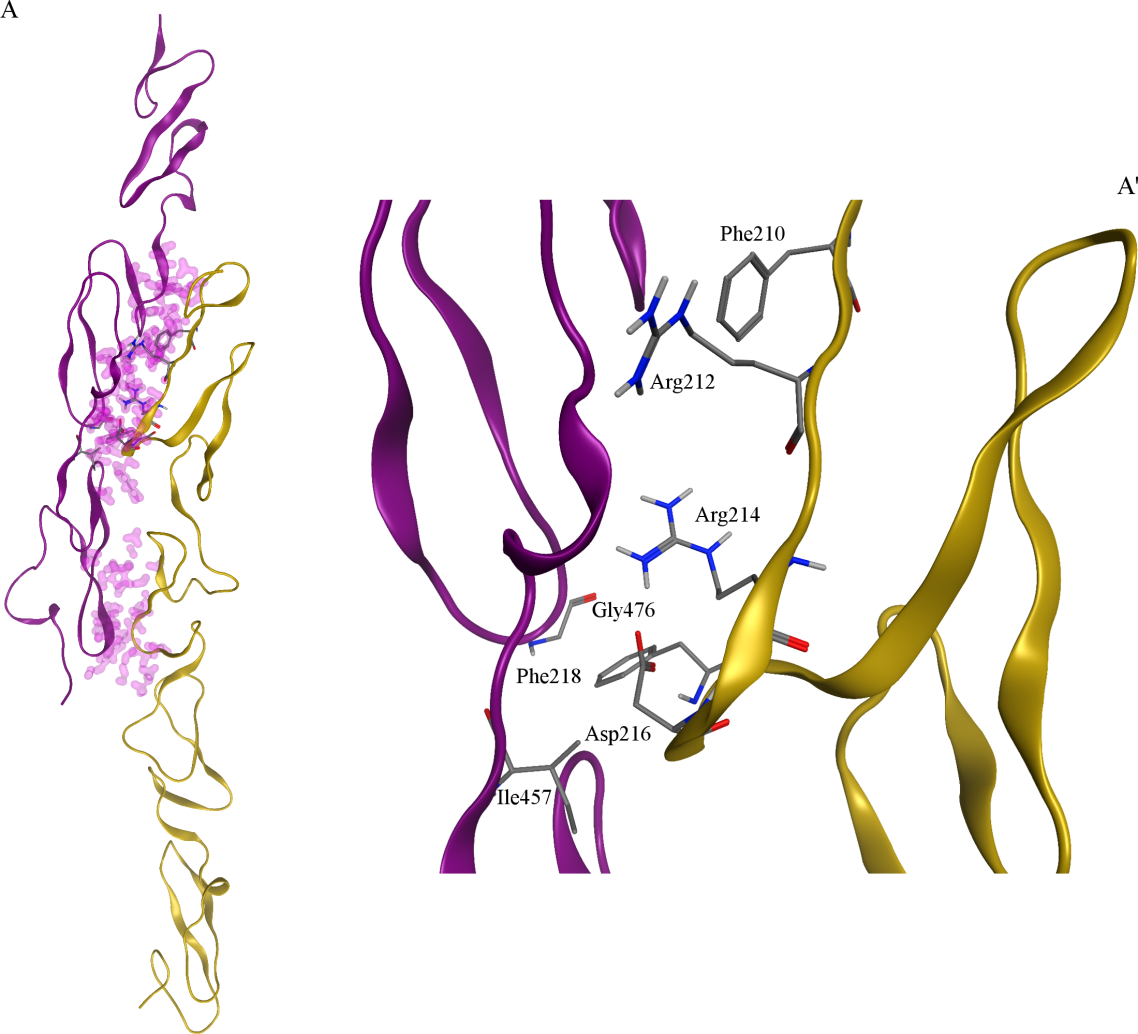
Notch2::Jagged2 complex. (A) Notch2::Jagged2 complex obtained by protein::protein docking followed by homology modelling, shown as whole representation. Notch2 is shown as purple ribbon; Jagged2 is shown as yellow ribbon. Residues involved in Notch2::Jagged2 contacts are highlighted by shells rendered according to van deer Waals distance. Conserved residues at the interface of the complex are shown in stick representation. (A’) Notch2::Jagged2 detailed view.

Notch2::Jagged2 complex was prepared by comparative modelling based on the use of the previous Notch1::Jagged1 complex as a template. The alignment at the basis of the modelling procedure was characterized by a very high number of identical residues: 53% Notch1 versus Notch2, and 51% Jagged1 versus Jagged2. The analysis of the Notch2::Jagged2 interaction surface, carried out with the support of the MOE ‘Protein contact tool, allowed us to retrieve the conserved residues Ile457, Gly476, for Notch2; Phe210, Arg212, Arg214, Asp216, Phe218 in Jagged2, corresponding to the same residues in Jagged1, as reported in Fig 1.

The interaction surfaces of Notch2::Jagged2 complex computed as Van der Waals accessible surface is reported in S2 Fig.

**Fig 2:**
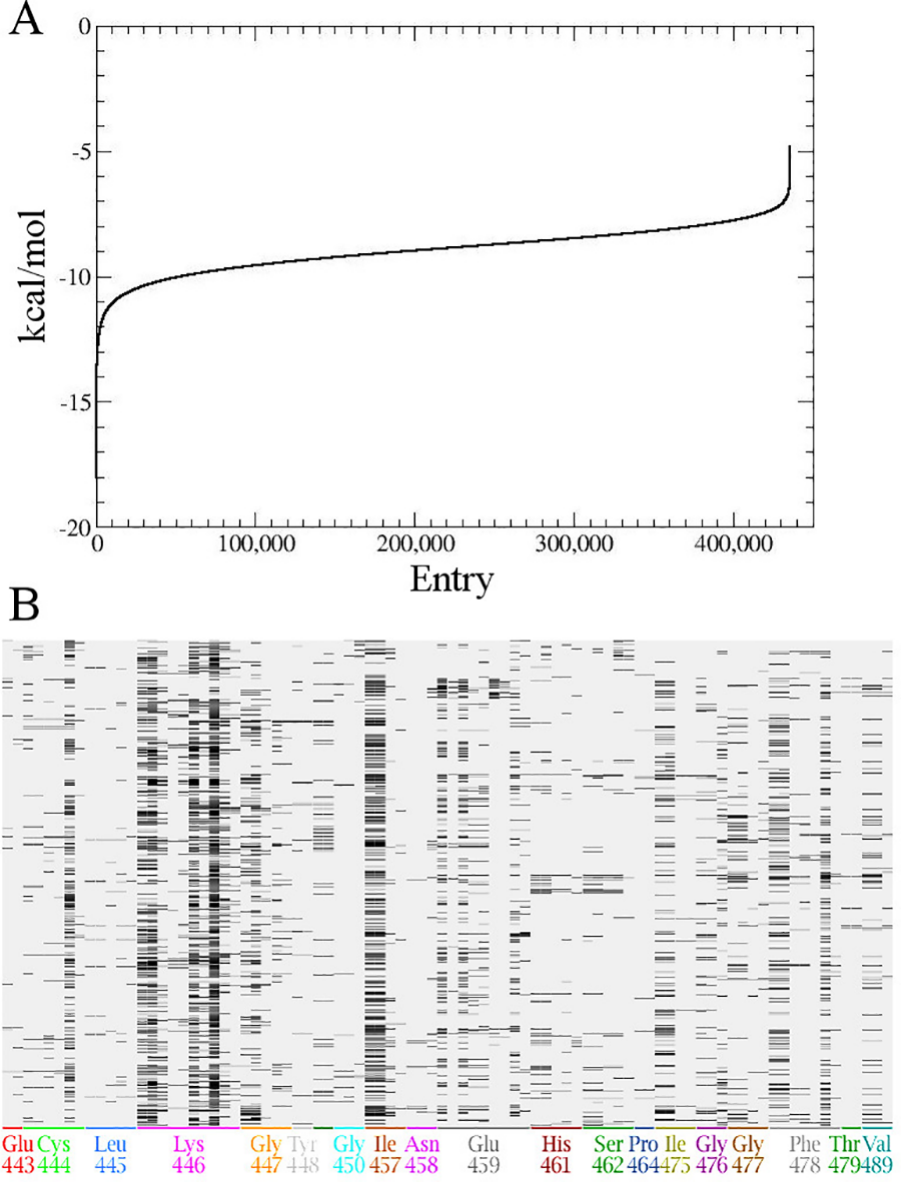
Virtual high-throughput screening on Notch2 interaction surface. (A) Plot of the docking score according to the London dG empirical scoring function for the resulting 435,835 compounds after the first virtual screening docking phase. The Y axis reports the docking score (kcal/mol) of the unique pose obtained for each tested entry (X axis). These values are sorted according to ascending docking score values along the Y axis. (B) Protein Ligand Interaction Fingerprint between Notch and the 30 retained docking poses for all the 25 best-scoring compounds tested in the second docking phase. The barcode display represents each docking pose (row) and protein::ligand fingerprints bit (column) as a matrix, in which a set bit is drawn as a black rectangle; the X-axis shows the residue that corresponds to each group of fingerprint bits, and is coded with an arbitrary sequence of colors.

Since our inhibitory strategy was focused on the identification of novel small molecules able to selectively disrupt Notch::Jagged interaction, in order to avoid unspecific interfering with the Notch-independent Jagged signaling [36,37], we decided not to use the structure of the complex, but to carry out the *in silico* HTS only on the Notch2 interaction surface. In the first docking step, 435,835 out the original 438,407-compound database were retained. Fig 2A reports the docking scores (kcal/mol) obtained after the first virtual screening phase for all 435,835 docked compounds, computed through the London dG empirical scoring function. In this first preliminary screening phase, these values have been used just for relatively ranking the tested compounds, since they are affected by a significant level of approximation.

The top-scoring 25 molecules define a curve characterized by high first derivative values, which reduce for subsequent compounds, so a binding free energy cut-off value of -15.75 kcal/mol was set for compound selection. The compounds with binding free energy above -15.75 (kcal/mol) were instead discarded. The 25 selected molecules were submitted to a more extensive docking procedure, in which the docking score for each protein::ligand complex was computed through the GBVI/WSA dG scoring function. This procedure allows for a more accurate estimation of the affinity (see Table 1).

**Table 1:** Binding free energy of the 25-top scoring compounds after HTS.

	Virtual Screening	Accurate docking
Compound	Binding free energy (kcal/mol)	Binding free energy (kcal/mol)
1	-15.95	-8.19
2	-15.85	-7.30
3	-15.77	-6.96
4	-16.03	-6.88
5	-16.66	-6.72
6	-15.74	-6.59
7	-17.10	-6.49
8	-18.04	-6.48
9	-16.08	-6.46
10	-15.97	-6.39
11	-17.11	-6.29
12	-16.18	-6.26
13	-15.83	-5.99
14	-16.77	-5.77
15	-16.37	-5.77
16	-16.03	-5.74
17	-16.42	-5.72
18	-16.44	-5.67
19	-15.78	-5.61
20	-15.91	-5.49
21	-16.12	-5.35
22	-15.77	-5.28
23	-15.86	-5.27
24	-15.77	-4.70
25	-17.36	-4.12
26	-8.04	-

Thirty poses for each of the top-scoring 25 compounds were analysed according to the Protein Ligand Interaction Fingerprint (PLIF) procedure (Fig 2B), and they were demonstrated to target the Notch2 residues already identified as the key partners in Notch1::Jagged1 interaction, used as a guide during the protein::protein docking procedure, as previously described.

A detailed map of the interaction types engaged by each residue after the accurate docking phase with the 25 top-scoring compounds are reported in Table 2. For an easier reading, only the best score for each ligand is reported. S3 Fig shows their two-dimensional (2D) chemical structure.

**Table 2:** Binding data of the 25 top scoring compounds after accurate docking phase.

entry	Glu443	Cys444	Leu445	Lys446	Gly447	Tyr448	Ala449	Gly450	Ile457	Glu459	His461	Ser462	Ile475	Gly476	Gly477	Phe478	Thr479	Val489
**1**	--	----	----	DA------	----	--	--	--	DA--	--A---C-	----	----	-A--	--	---	DA--C-	--	--
**2**	D-	--daC-	---R	DA--I-C--	daa--	--	DA	--	DA--	D-A----	----	----	---	--	---	-----	--	--
**3**	D-	---C-	----	DA----C--	----	--	--	-a-	DA--	------R	----	--AA	---	--	---	-----	--	--
**4**	--	---C-	----	-----CR-	--RR	--	--	--C	---	------	----	----	---	--	---	-----	--	--
**5**	-C	D--C-	----	DA--I-C--	----	--	--	--	DA--	------	----	----	DA--	--	---	-----	--	--
**6**	--	---C-	----	-----CR-	--R-	--	--	--	DA--	------	----	----	---	--	da--	----CR	--	DA-
**7**	--	----	----	-------	da--	--	--	--	---	D-A----	----	----	DA--	--	---	DA--CR	--	DA-
**8**	--	---C-	----	DA--I-CR-	da-RR	da	--	--	DA--	------	----	----	DA--	--	---	-----	--	--
**9**	--	DA--C-	----	DA----C--	----	--	--	--	---	------	----	----	DA--	--	---	-----	--	--
**10**	--	----	----	-AA---C--	da--	--	--	--	DA--	D-Ada---	----	----	DA--	--	---	-----	--	--
**11**	--	----	----	-AA--IIC--	-a--	--	--	--	---	------	----	----	---	--C	da--	----C-	--	DA-
**12**	DC	---C-	----	DA------	da--	--	--	--	DA--	------	----	----	DA--	da-	---	-----	--	--
**13**	--	----	----	-------	da--	--	--	--	DA--	------	----	----	DA--	--	da--	--da-C-	--	DA-
**14**	--	----	----	-----CRR	----	--	--	--	DA--	D-A---CR	----	----	---	--	da--	DA----	--	--
**15**	--	----	----	DA----C--	da--	--	--	--	---	D-A---C-	----	----	DA--	daC	---	DA----	--	--
**16**	--	----	----	-A--I-C--	-aa--	--	--	--	DA-R	------	----	----	---	--	---	-----	--	--
**17**	--	----	----	-------	----	--	--	--	---	D------	----	----	---	d--	---	DAd--C-	DA	--C
**18**	--	---C-	----	----I-C--	----	--	--	--	---	------	----	----	DA--	--	---	-----	--	--
**19**	--	----	----	DA------	da--	--	--	--	DA--	---a--C-	----	----	---	--	---	-----	--	--
**20**	--	----	----	DA----CR-	----	--	--	--	DA--	------	----	----	---	--	---	-----	--	--
**21**	--	----	----	---a----	da--	--	--	--	DA--	-----CR	DAda-	--da-	---	--	---	-----	--	--
**22**	--	----	----	----I-C--	-aa--	--	--	--	DA--	D-A----	----	----	DAda	da-	---	----C-	--	--
**23**	--	----	----	-------	da--	--	--	--	DA--	------R	----	----	DA--	--	---	--aaC-	--	DA-
**24**	--	----	----	-AA--IIC--	----	--	--	--	DA--	------	----	----	---	--	---	-----	--	--
**25**	--	----	----	DA----C--	----	--	--	--	---	------	----	----	---	--	---	-----	--	--

Legend -: bit not set, D: sidechain hydrogen bond donor; A: sidechain hydrogen bond acceptor; d: backbone hydrogen bond donor; a: backbone hydrogen bond acceptor; I: ionic attraction; C: surface contact; R: arene attraction.

As shown in Fig 2B and in Table 2, all the 25 top-scoring ligands are involved in a diffuse set of polar and non-polar interactions with the surrounding resides, among which Cys444, Lys446, Gly447, Ile457, Gly777 seem to be the most relevant ones.

All the 25 top-scoring poses were carefully analyzed and the 2D ligand interaction plot was generated and summarized in Table 2.

**Fig 3:**
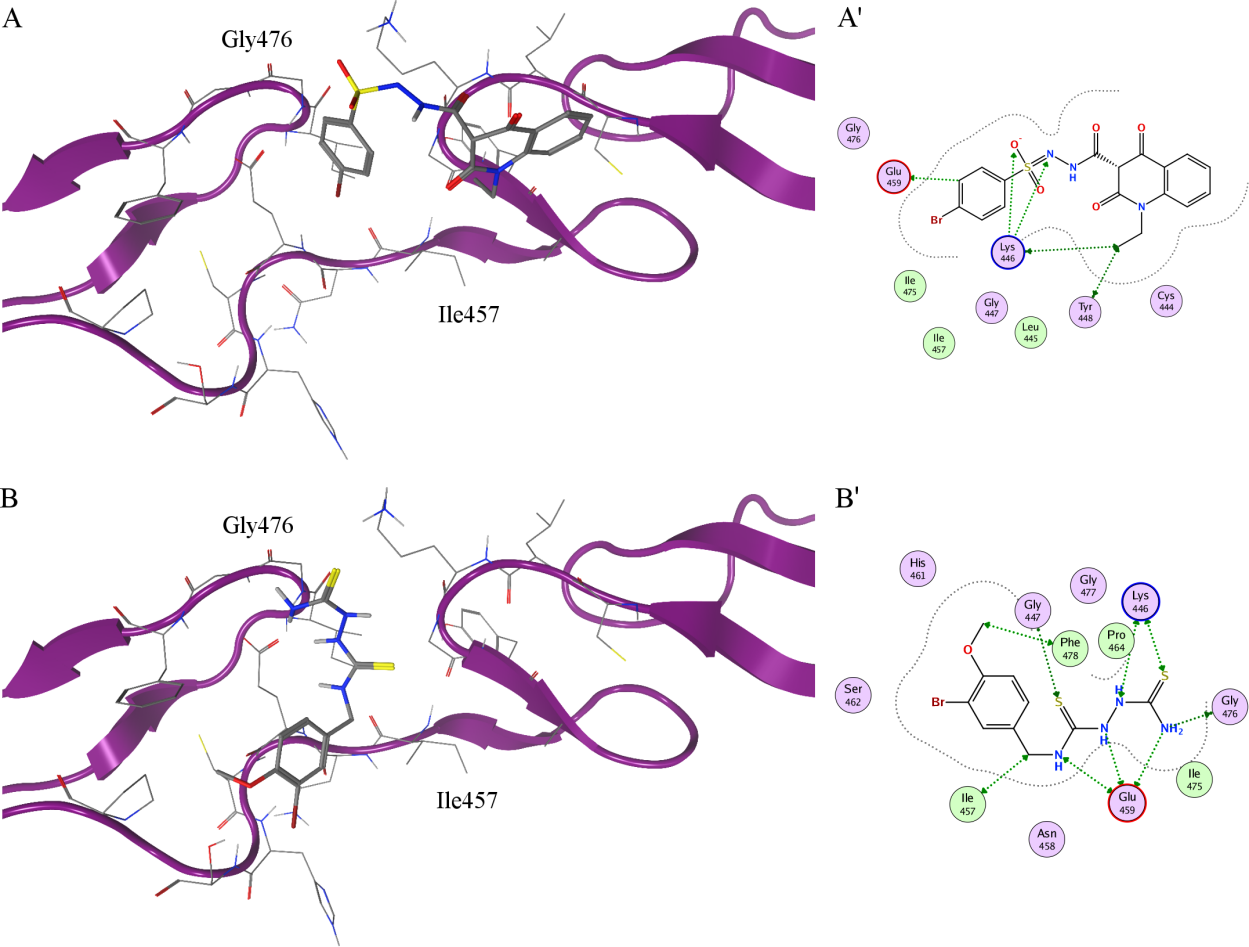
*In silico* complexes between Notch2 and the IGOR compounds. (A, B) Best docking poses of the IGOR compounds, selected through virtual HTS on Notch2 homology model. Notch2 is shown as ribbon; residues in the active site are shown in stick representation; IGOR1 and IGOR2 are shown in stick representation in A and B, respectively. (A’, B’) Two-dimensional ligand interaction scheme of IGOR compounds. Polar and greasy residues are shown in lilac and lime respectively; acidic and basic residues are highlighted by red and blue contours, respectively. Residues involved in protein::ligand interactions are highlighted by green arrows; proximity contour by dotted lines.

Two compounds, named IGOR1 and IGOR2, able to specifically target the Notch2 residues engaged by the Jagged2 interaction, were selected and submitted to a final refinement procedure with LigX, allowing the relaxation of the complex, through a tethered refinement of the protein binding site and an *in situ* MM-based ligand optimization. The optimized ligand::protein complexes obtained for the selected compounds are reported in Fig 3A and 3A’, for IGOR1, and in Fig 3B and 3B’, respectively. This approach allowed us to compute a more accurate binding free energy and K_d_ value for the two selected compounds: -4.96 kcal/mol and 223.8 μM for IGOR1, and -5.94 kcal/mol and 42.5 μM for IGOR2, respectively.

The two IGOR compounds were evaluated for their biological efficacy.

### Biological effect of selected IGOR compounds

**Fig 4:**
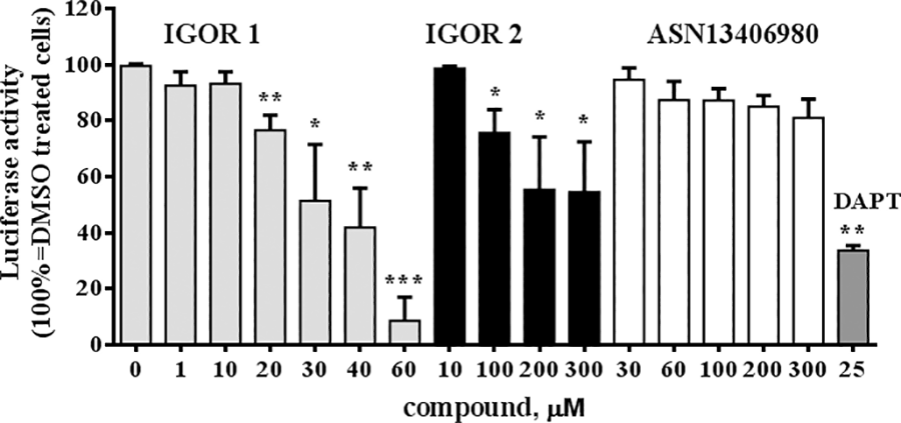
Inhibitory effect of the IGOR compounds on Notch transcriptional activity. A Notch-responsive luciferase assay was carried out on HEK293T cells treated with different concentrations of IGOR1 and IGOR2, the positive and negative controls DAPT and ASN13406980, respectively. After 48 h of incubation luciferase activity was measured by dual luciferase reporter assay. Data are expressed as mean value ± SD of three experiments. Statistical analysis was performed by unpaired two-tailed Student’s t-test. *** ^=^ *p* < 0.001; ** ^=^ *p* < 0.01; * ^=^ *p* < 0.05.

We evaluated the ability of 2 out of 438,407 tested compounds to inhibit Notch activity by measuring changes in Notch transcriptional activity through a Notch-responsive gene reporter assay (Fig 4). The Notch-responsive assay allows to measure its transcriptional activity through ICN-CSL association using a reporter plasmid containing a firefly luciferase gene under the control of multimerized (6x) CSL responsive element upstream of a minimal promoter. HEK293T cells were transfected with the 6xCSL plasmid and pRL-TK (to normalize for the transfection efficiency) and treated with IGOR1 and IGOR2 for 48 h. The screening concentrations were set up starting from the computational dissociation constants (K_d_) and within a concentration range that allowed to maintain the solvent concentration (DMSO) minor than 1%, to avoid any toxic effect. As a positive control of Notch activity inhibition, we used the γ-Secretase inhibitor, DAPT 25 μM. As a negative control, among the whole database of docked compounds, we selected a representative compound (ASN13406980, entry number 26 in Table 1) that, according to our *in silico* predictions, was assumed not to interfere with Notch2 due to its low affinity. Its binding free energy value was indeed significantly lower than that computed for IGOR1 and IGOR2 (in Table 1, entry number 8 and 1, respectively).

IGOR1 and 2 significantly reduced Notch transcriptional activity in a dose-dependent manner (Fig 4). IGOR1 exerted an inhibitory effect with an IC_50_ (33.75 ± 0.28 μM) lower than IGOR2 (IC_50_ 283.00 ± 29.52 μM). In particular, the effect of 30 μM IGOR1 displayed an efficacy similar to 25 μM DAPT.

Computational (affinity for Notch2) and experimental results (ability to uncouple Notch2::Jagged2 interaction) cannot be compared, since the ability of uncoupling Notch2::Jagged2 interaction is not only and directly connected to the affinity of these Notch2 ligands, but also depends on other structural and physico-chemical properties that influence their ability to unfavour the Notch2::Jagged2 molecular recognition.

On the other side, we believe that further steps of chemical refinement could be useful to increase the potency of IGOR2, whose IC_50_ is currently too high to exclude the presence of a toxic non-specific effect.

**Fig 5:**
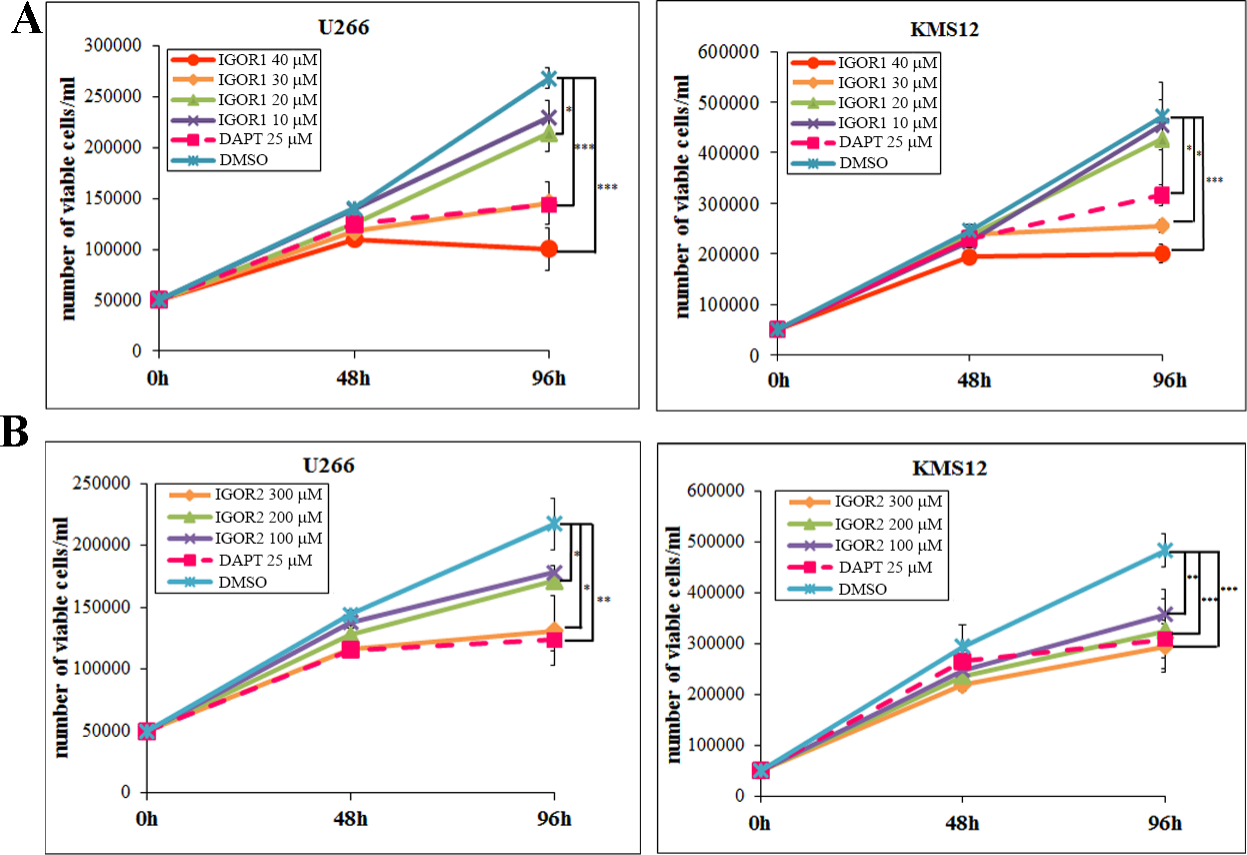
Effect of the IGOR compounds on MM cell viability. (A, B) Cell growth assay of U266 and KMS12 cells upon treatment with IGOR1 and IGOR2. Viability of MM cells was assessed by the volumetric count function of the FACS Verse BD flow cytometer. The values are mean ± SD. Statistical analysis was performed by unpaired two-tailed Student’s t-test. *** ^=^ p < 0.001; ** ^=^ p < 0.01; * ^=^ p < 0.05.

The effect of the selected compounds on cell viability was assessed. Cell growth assay was performed on two multiple myeloma cell lines, U266 and KMS12. These cells carry the dysregulated Notch pathway mainly due to a high expression of Notch2 receptors, and Jagged ligands; specifically, U266 cells exclusively express Notch2 and KMS12 cells express both Notch 1 and Notch2, with a prevalence of the second [24,25,38,39]. Therefore, these cells represent a suitable model to evaluate the biological outcome of Notch2 activation blockade induced by our compounds. Cell viability was evaluated, through flow cytometry, after 48h and 96h of incubation with different concentrations of IGOR1 and IGOR2 (Fig 5). We observed that the treatment with 30 μM and 40 μM IGOR1 for 48 h and 96 h induced a dose-dependent inhibition of cancer cell growth, respectively 45.8% and 62.6%, for U266 cells and 46.0% and 57.6%, for KMS12 cells at 96 h. This effect was comparable to that of DAPT (respectively 46.4% and 33.1% cell growth for U266 and KMS12 cells at 96 h). IGOR2 was less effective and showed an inhibitory effect at higher concentrations: 300 μM IGOR2 induced a cell growth inhibition of 39.7% in U266 cell line and 39.1% in KMS12 cells.

On the whole, the inhibitory effect on cancer cell viability of the two compounds directed specifically to inhibit the activation of Notch receptors triggered by Jagged ligands was comparable to that of GSI. This is in accordance with our previous results indicating that Notch signaling inhibition obtained by silencing Jagged1 and 2 results in decreased U266 cell growth [17] and osteoclastogenic potential [22].

## Conclusions

This work represents an efficient and integrated strategy spanning *in silico* and *in vitro* approaches useful to identify, test and validate novel compounds able to disrupt the Notch::Jagged interaction, switching off the subsequent signalling pathway.

The use of molecular modelling tools allowed us to specifically target, at an atomistic level, the Notch residues involved in the Notch::Jagged molecular recognition mechanism, without perturbing the activation of Notch pathways not mediated by Jagged, and without altering the other Jagged functions [36,37].

Comparative modelling procedures applied to the structural study of Notch has been validated *in vitro* by the identification of active IGOR compounds able to inhibit the Notch signalling pathway. In this paper, we mainly focused our attention on Notch2, which plays a key role in multiple myeloma [17,21,22] and other hematological malignancies, including splenic marginal zone lymphoma [40], B-cell chronic lymphocytic leukemia [41] and diffuse large B-cell lymphomas [42] as well as in other solid cancers including glioma [43], lung adenocarcinoma [44], oesophageal squamous cell carcinoma [45], cervical cancer [46], hepatocellular carcinoma [47], gastric cancer [48] and pancreatic carcinoma [49]. Even if, at the moment, no molecular data about the selectivity of IGOR compounds with respect to the different Notch isoforms are available, the growth inhibition of U266 cells, which carry exclusively Notch2, suggested the IGORs ability to target this isoform.

These results lay the foundations for the development of an integrated pipeline to further identify selective Notch inhibitors through a differential comparative modelling of all the Notch isoforms suitable for improving the proposed pipeline. If selective compounds will be identified, this approach promises to overcome gut toxicity induced by common pan-Notch inhibitory drugs, i.e. GSIs. Indeed, a recent report indicates that intestinal toxicity can be avoided through the selective inhibition of Notch activation mediated only by Jagged ligands or alternatively by Dll ligands [19].

The present approach provides the proof of concept that it is possible to target Notch::Jagged interaction *via* small molecules, and poses the bases for future pharmacological strategies aimed at operating on this signaling pathway by an unprecedented strategy. Indeed, small molecules have several advantages with respect to biotechnological drugs, since they can be easily delivered, are metabolically stable and orally active. Additionally, their administration does not need inpatient treatment thereby improving patients’ quality of life and reducing social costs.

## Supporting information

S1 Fig *In silico* high-throughput screening pipeline. (TIFF)

S2 Fig Notch2::Jagged2 interaction surface. (A) Notch2 and (B) Jagged2 interaction surface computed as van der Waals accessible surface. The surface is coloured according to the lipophilic potential calculated from the Wildman and Crippen SlogP parameters: hydrophobic regions are shown in green, hydrophilic regions in purple and neutral regions in white.
(TIFF)

S3 Fig 2D chemical structures of the 25 top-scoring compounds selected through *in silico* HTS on Notch1. (TIFF)
