# Supplementary figures and images for: Identification of small molecules uncoupling the Notch::Jagged interaction through an integrated high-throughput screening
Source: PLoS One. 2017 Nov 3;12(11):e0182640. doi: 10.1371/journal.pone.0182640 (PMC5669421; doi:10.1371/journal.pone.0182640)

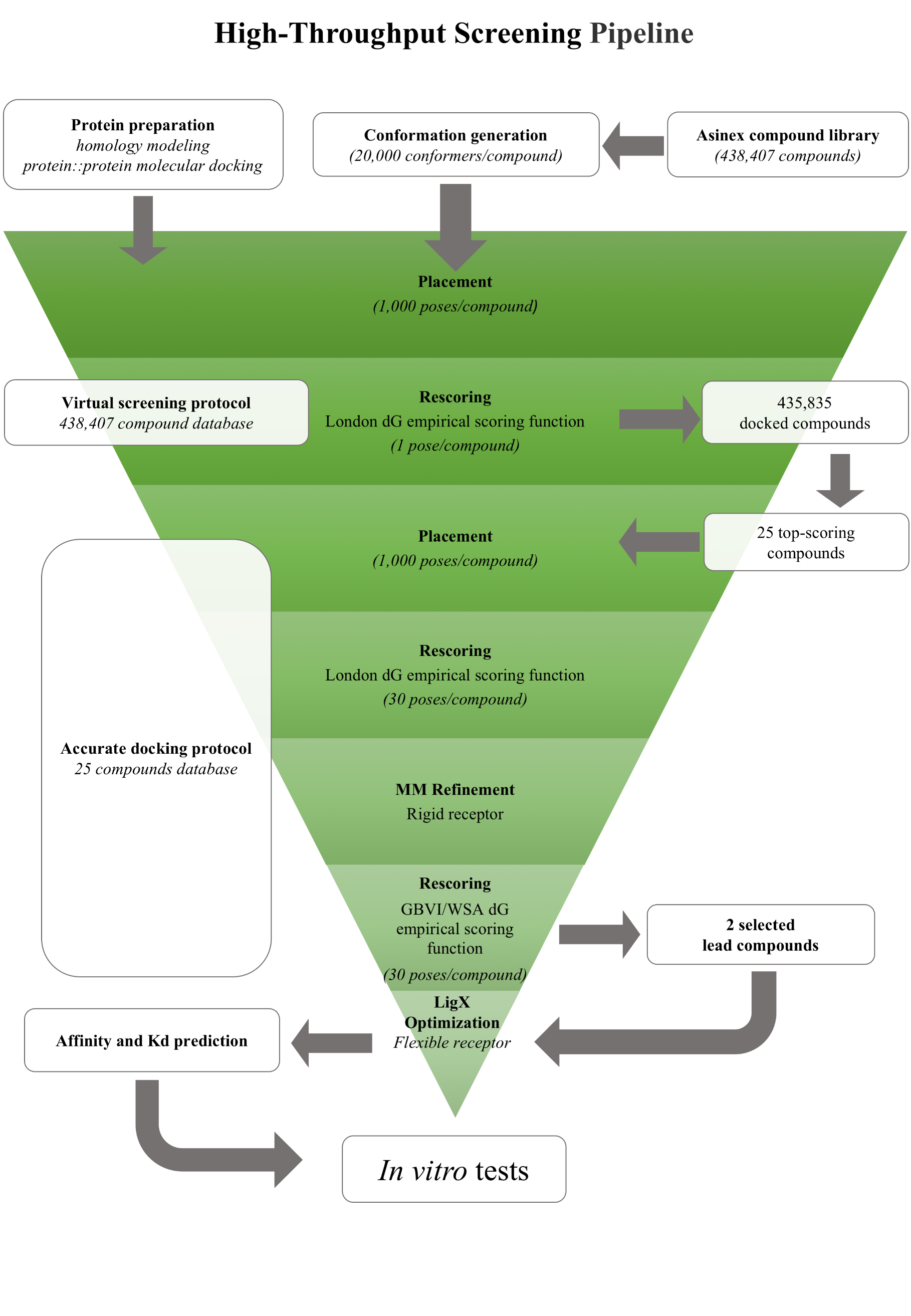

Supplement: S1 Fig — (TIFF) [file pone.0182640.s001.tiff]

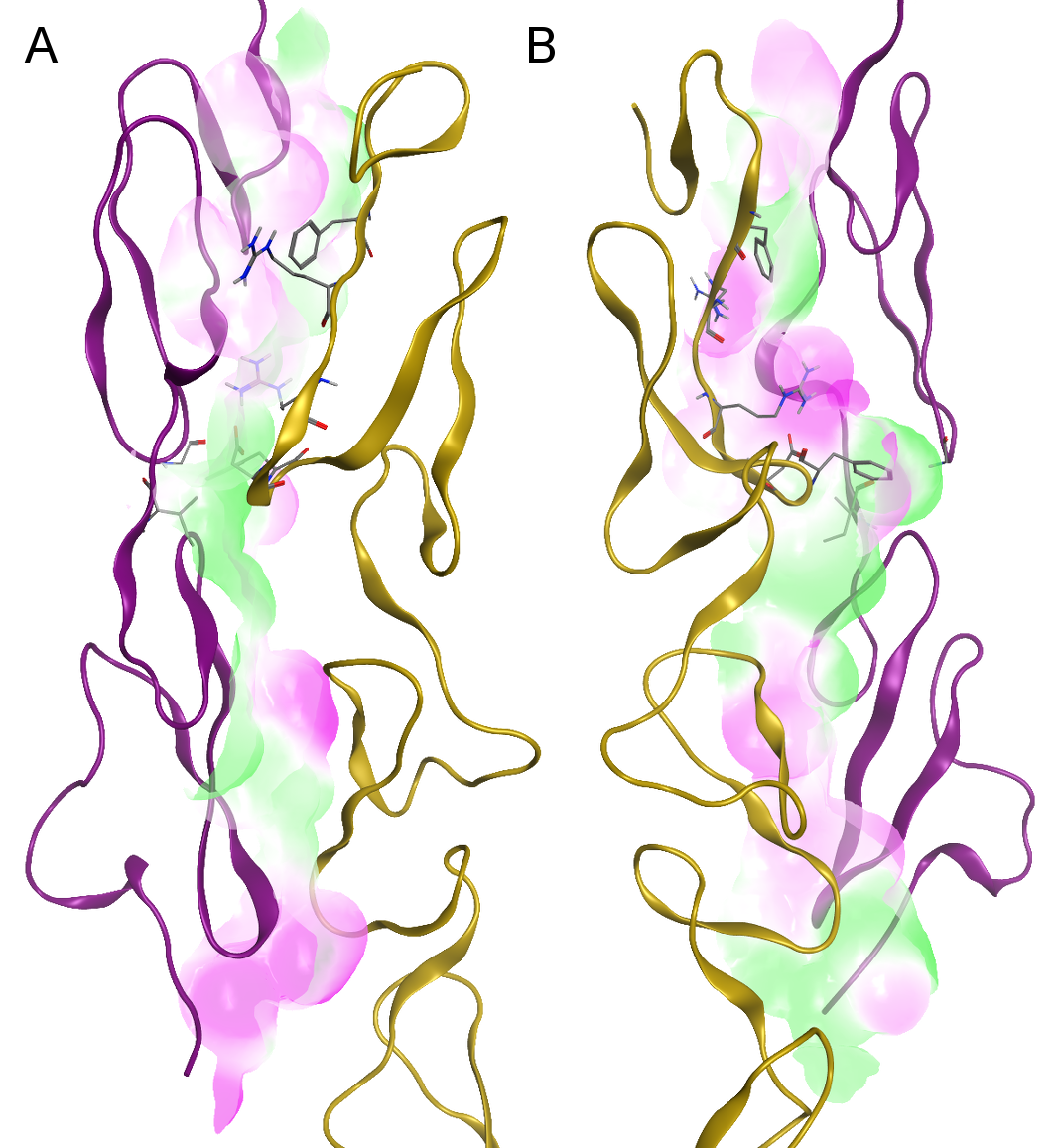

Supplement: S2 Fig — (A) Notch2 and (B) Jagged2 interaction surface computed as van der Waals accessible surface. The surface is coloured according to the lipophilic potential calculated from the Wildman and Crippen SlogP parameters: hydrophobic regions are shown in green, hydrophilic regions in purple and neutral regions in white. (TIFF) [file pone.0182640.s002.tiff]

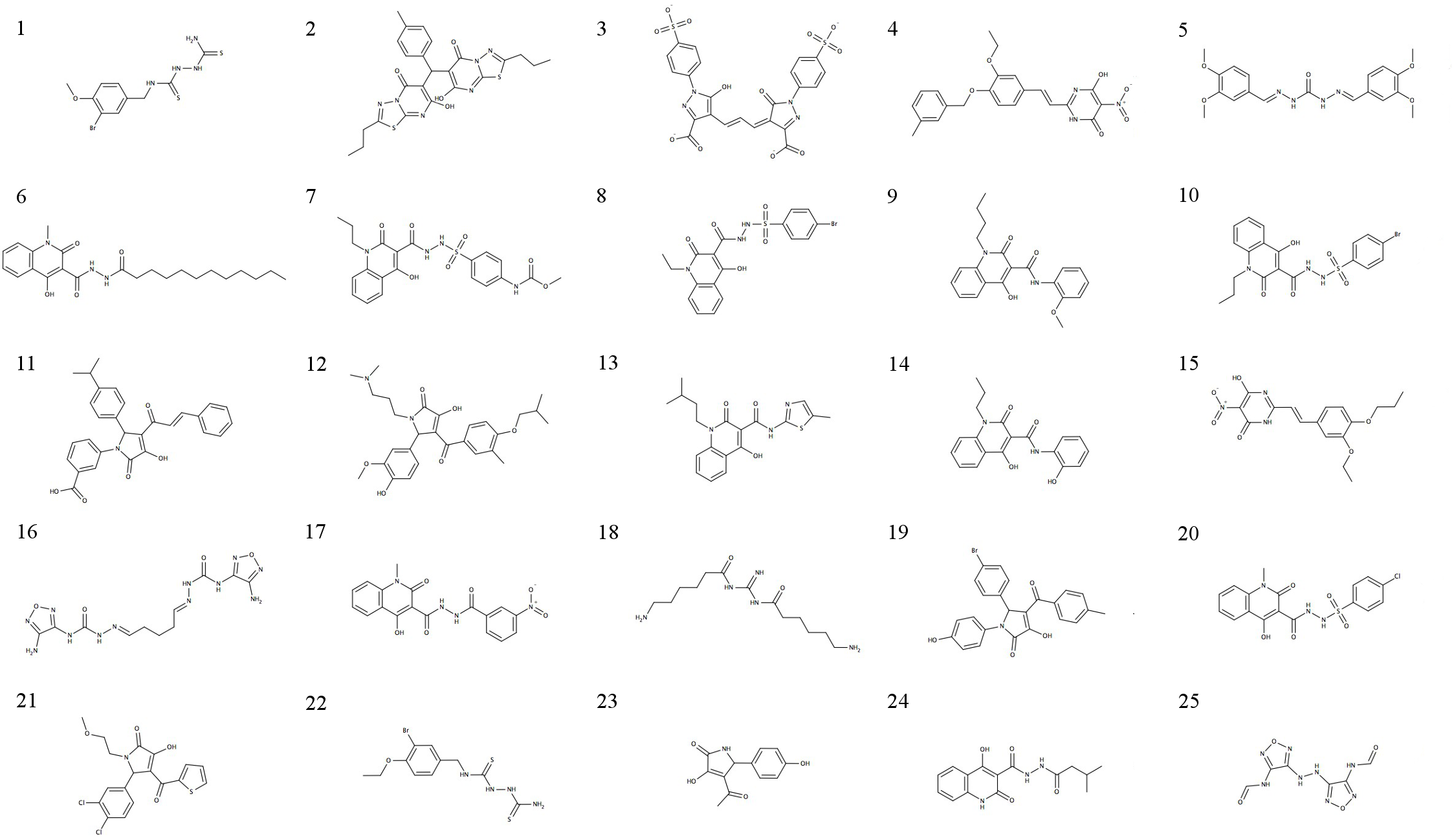

Supplement: S3 Fig — (TIFF) [file pone.0182640.s003.tiff]
